# Healthcare professionals' perceived barriers and facilitators of health behavior support provision: A qualitative study

**DOI:** 10.1002/cam4.5445

**Published:** 2022-11-17

**Authors:** Eline Bouwman, Saskia M. F. Pluijm, Iridi Stollman, Vera Araujo‐Soares, Nicole M. A. Blijlevens, Cecilia Follin, Jeanette Falck Winther, Lars Hjorth, Tomas Kepak, Katerina Kepakova, Leontien C. M. Kremer, Monica Muraca, Helena J. H. van der Pal, Carina Schneider, Anne Uyttebroeck, Gertrui Vercruysse, Roderick Skinner, Morven C. Brown, Rosella P. M. G. Hermens, Jacqueline J. Loonen, the PanCareFollowUp Consortium

**Affiliations:** ^1^ Department of Hematology, Radboud Institute for Health Sciences Radboud University Medical Center Nijmegen The Netherlands; ^2^ Princess Máxima Centre for Pediatric Oncology Utrecht The Netherlands; ^3^ Department of Health Technology & Services Research, Technical Medical Center University of Twente Enschede The Netherlands; ^4^ Oncology, Department of Clinical Sciences Lund Lund University, Skåne University Hospital Lund Sweden; ^5^ Childhood Cancer Research Group Danish Cancer Society Research Center Copenhagen Denmark; ^6^ Department of Clinical Medicine Aarhus University and Aarhus University Hospital Aarhus Denmark; ^7^ Pediatrics, Department of Clinical Sciences Lund Lund University, Skåne University Hospital Lund Sweden; ^8^ International Clinical Research Center (FNUSA‐ICRC) at St. Anne's University Hospital Masaryk University Brno Czech Republic; ^9^ Department of Pediatrics, Emma Children's Hospital Amsterdam UMC Amsterdam The Netherlands; ^10^ Faculty of Medicine Utrecht University and Utrecht Medical Center Utrecht the Netherlands; ^11^ DOPO Clinic, Division of Pediatric Hematology and Oncology IRCCS Istituto Giannina Gaslini Genoa Italy; ^12^ PanCare Bussum The Netherlands; ^13^ Childhood Cancer International—Europe Vienna Austria; ^14^ Department of Oncology, Pediatric Oncology, KU Leuven, Department of Pediatric Hematology and Oncology University Hospitals Leuven Leuven Belgium; ^15^ Wolfson Childhood Cancer Research Center, Newcastle University Centre for Cancer Newcastle University Newcastle upon Tyne UK; ^16^ Great North Children's Hospital Royal Victoria Infirmary Newcastle upon Tyne UK; ^17^ Wolfson Childhood Cancer Research Center, Translational and Clinical Research Institute Newcastle Universtiy Newcastle upon Tyne UK; ^18^ Population Health Sciences Institute Newcastle University Newcastle upon Tyne UK; ^19^ Scientific Institute for Quality of Healthcare (IQ Healthcare), Radboud Institute for Health Sciences Radboud University Medical Center Nijmegen The Netherlands

**Keywords:** clinical management, pediatric cancer, screening, survival

## Abstract

**Background:**

Childhood cancer survivors (CCSs) have an increased risk of developing chronic health conditions. Evidence suggests that poor health behaviors further increase health risks. Healthcare professionals (HCPs) involved in survivorship care have a key role in providing health behavior support (HBS) but can feel limited in their ability to do so. This study aims to explore European HCPs perceived facilitators and barriers to providing HBS to CCSs.

**Methods:**

Five focus groups with 30 HCPs from survivorship care clinics across Europe were conducted. Topic guides were informed by the Theoretical Domains Framework (TDF) to capture domains that may influence provision of HBS. Focus groups were analyzed with thematic analysis. Transcripts were inductively coded, after which axial coding was applied to organize codes into categories. Finally, categories were mapped onto the TDF domains.

**Results:**

Nine TDF domains were identified in the data. The most commonly reported TDF domains were “Knowledge”, “Skills”, and “Environmental context and resources”. HCPs indicated that their lack of knowledge of the association between late effects and health behaviors, besides time restrictions, were barriers to HBS. Facilitators for HBS included possession of skills needed to pass on health behavior information, good clinic organization, and an established network of HCPs.

**Conclusions:**

This study identified education and training of HCPs as key opportunities to improve HBS. Survivorship care clinics should work towards establishing well‐integrated structured care with internal and external networks including HBS being part of routine care. Proper understanding of facilitators and barriers should lead to better survivorship care for CCSs.

## Graphical abstract

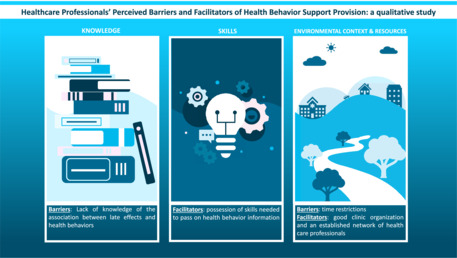

## 1 INTRODUCTION

Over the last 50 years, survival from childhood cancer has tremendously increased. Consequently, the childhood cancer survivor (CCS) population in Europe and the United States currently consists of over 300,000 and 400,000 people, respectively.^1^, ^2^, ^3^, ^4^ However, CCSs are at increased risk of developing chronic health problems (late effects), resulting in excess morbidity and mortality.^5^, ^6^, ^7^, ^8^

Unhealthy behaviors in CCSs, such as physical inactivity, poor diet, smoking, excessive alcohol consumption, and drug use, further increase the risk of developing late effects.^9^, ^10^ As CCSs are already vulnerable to poor health, cancer organizations and guidelines developed for childhood cancer survivors recommend CCSs to comply with healthy behaviors.^10^, ^11^, ^12^ However, a high proportion of CCSs do not meet these recommendations.^13^, ^14^, ^15^, ^16^ This is illustrated in a study by Robien et al. in which dietary index scores were relatively low in survivors of childhood acute lymphoblastic leukemia, indicating poor adherence to recommended dietary guidelines.^13^ Robien suggests the lack of survivor‐specific dietary guidelines to guide clinicians, survivors, and caregivers of key nutrition messages to be a potential reason for this low adherence. Furthermore, a study by Ness et al. showed that only 46% of CCSs met physical activity recommendations with survivors who received cranial radiation or underwent an amputation being at high risk for physical inactivity.^15^

Health behavior support (HBS) in the cancer survivorship care setting, consisting of screening for unhealthy behaviors, as well as informing and advising CCSs about adopting healthier behaviors, may help CCSs in adopting these behaviors. Considering the key role healthcare professionals (HCPs) have when providing HBS to CCSs, it is crucial to understand the current barriers and facilitators to HBS provision. For instance, HCPs may not be aware of the role of healthy behaviors in decreasing the risk of developing late effects of cancer.^17^, ^18^ In addition, HCPs may experience barriers at a personal or organizational level, which may hinder their efforts to provide HBS for CCSs. Exploring these will help future implementation of health behavior support in current practice and interventions designed for CCSs.^19^, ^20^

**FIGURE 1:**
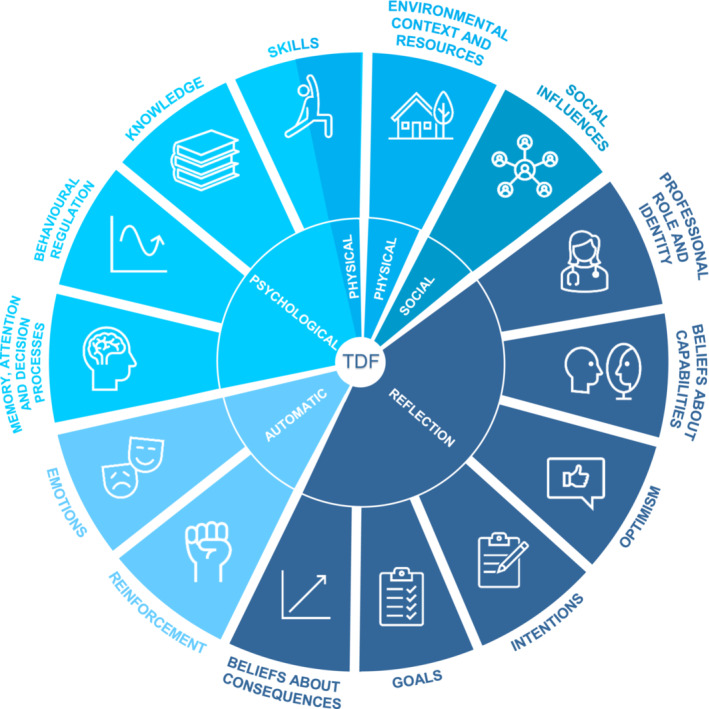
Domains of the Theoretical Domains Framework—adapted from Cane et al.^21^

One way to have a comprehensive understanding of barriers and facilitators to HBS provision by HCPs in the survivorship care setting, is by using the Theoretical Domains Framework (TDF) as a guidance (Figure 1, Table 1).^21^, ^22^ The TDF is a theoretical framework resulting from the synthesis of 128 constructs from 33 behavior change theories. These 128 constructs were aggregated, by experts, into 14 domains that aim at explaining behavior and behavior change. These capture either psychological, physical, social, reflective, or automatic influences on behavior implementation. Understanding the factors that determine clinical behavior can be used to inform the design of behavior change interventions.

**TABLE 1:** The Theoretical Domains Framework (v2) domains and definitions^20^, ^21^

Knowledge: an awareness of the existence of something
2 Skills: an ability or proficiency acquired through practice
3 Social/professional role and identity: a coherent set of behaviors and displayed personal qualities of an individual in a social or work setting
4 Beliefs about capabilities: acceptance of the truth, reality, or validity about an ability, talent, or facility that a person can put to constructive use
5 Optimism: the confidence that things will happen for the best or that desired goals will be attained
6 Beliefs about consequences: acceptance of the truth, reality, or validity about outcomes of a behavior in a given situation
7 Reinforcement: increasing the probability of a response by arranging a dependent relationship, or contingency, between the response and a given stimulus
8 Intentions: a conscious decision to perform a behavior or a resolve to act in a certain way
9 Goals: mental representations of outcomes or end states that an individual wants to achieve
10 Memory, attention and decision processes: the ability to retain information, focus selectively on aspects of the environment and choose between two or more alternatives
11 Environmental context and resources: any circumstance of a person's situation or environment that discourages or encourages the development of skills and abilities, independence, social competence, and adaptive behavior
12 Social influences: those interpersonal processes that can cause individuals to change their thoughts, feelings, or behaviors
13 Emotion: a complex reaction pattern, involving experiential, behavioral, and physiological elements, by which the individual attempts to deal with a personally significant matter or event
14 Behavioral regulation: anything aimed at managing or changing objectively observed or measured actions

This study aims to explore facilitators and barriers to HBS as perceived by HCPs in European survivorship care centers. Given that this is the first exploratory study within this area, the decision was made to use the TDF as within this framework all potential factors that influence human behavior are accounted for.

## 2 METHODS

### 2.1 Design and setting

This explorative qualitative study was part of the European‐wide PanCareFollowUp project and used in‐depth focus groups. The study was carried out according to the standards for reporting qualitative research (SRQR).^23^ The procedures were approved by METC Oost‐Nederland (case number 2019–5630).

The setting for the focus groups with HCPs included survivorship care centers in Belgium (center 1), the Czech Republic (center 2), Sweden (center 3), and the Netherlands (centers 4 and 5). All centers were awarded with a Horizon 2020 grant to participate in the PanCareFollowUp‐project.

### 2.2 Participants and recruitment

A convenience sampling method was applied to recruit HCPs (e.g., oncologists, nurses, physiotherapists, etc.) involved in childhood cancer survivorship care and in the possession of sufficient English or Dutch language skills. Each participating center approached and informed HCPs about the study. Subsequently, HCPs could register their interest to the research team via email. All focus groups aimed to recruit 5 to 10 participants with at least one nurse and (pediatric) oncologist/internist present to ensure representative data. In this study, the sample size was dependent on the centers participating in the PanCareFollowUp project, which led to a specific number of focus groups (number of participating centers; *n* = 5). Nevertheless, due to the narrow study aim and specificity of this study, this number is thought to be adequate to reach sufficient information power and code saturation.^24^, ^25^ To reflect on data saturation, repetition of observed patterns in the data was evaluated after the fifth focus group. Written informed consent was obtained from all participants.

### 2.3 Procedures

The focus groups were facilitated by a female PhD‐candidate (EB), an experienced qualitative researcher with an interest in health behaviors of CCSs. A note‐taker took field notes of key points raised by the participants during the focus groups (RH, IS, or SP).

The focus groups took place between September 2019 and April 2020. Three focus groups were conducted on‐site at the centers, whereas, due to COVID‐19 restrictions and researchers being unable to travel to the clinic, two focus groups took place via a videoconference application. At the start of each focus group, participants completed a sociodemographic questionnaire to capture relevant background information such as age, profession, and number of years of working experience in (pediatric) oncology.

The focus groups followed a semi‐structured topic guide and started with general open‐ended questions on HBS in current survivorship care (Table S1). Thereafter, HCPs were asked about possible facilitators and barriers to providing HBS to CCSs. Given the exploratory nature of this study, the TDF was used to inform the topic guide as it incorporates all potential factors that may influence human behavior^21^, ^22^ (Figure 1, Table 1). Focus groups were audio‐recorded, transcribed verbatim, and lasted approximately 45–60 minutes. Transcripts were anonymized for analysis.

### 2.4 Analysis

**FIGURE 2:**
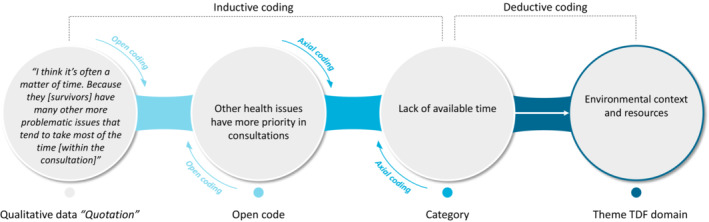
Illustrative example of the coding process.

Data were analyzed in Atlas.ti 8.3.20 for Windows by applying thematic analysis to look for recognizable topics and patterns in the data and by using the TDF as a theoretical framework.^21^, ^22^, ^26^ First, after familiarization with the transcripts of the focus groups, two researchers (EB, IS) independently created open codes on a sentence level using an inductive approach in an iterative process (Figure 2). After coding each transcript, EB and IS discussed the transcript to reach a consensus about the open codes. Next, axial coding was applied by organizing and grouping open codes to compile categories.^27^ Thereafter, to conceptualize these categories regarding different types of influences on behaviors, the categories were deductively mapped into the predefined TDF domains (Figure 1, Table 1).^21^, ^22^ A TDF domain was considered relevant for this study if during the focus group facilitators or barriers to HBS concerning the TDF domain were mentioned. Any discrepancies in the analysis were discussed among the researchers until consensus was reached. If needed, a third person could be consulted to resolve discrepancies.

## 3 RESULTS

### 3.1 Sample characteristics

In total, five focus groups were conducted among HCPs. Though the number of focus groups was decided a priori, after the five focus groups, the observed patterns in the data were repeating, meaning that we had reached data saturation. Table 2 outlines the participant characteristics by center. Most participants were female, aged between 40–60 years old with up to 40 years of working experience in pediatric oncology. The sample comprised mostly of (pediatric) oncologists/internists and nurses. The participating centers see on average between 18 and 200 CCSs per month in their survivorship care clinic.

**TABLE 2:** Sample characteristics of the HCPs participating in focus groups displayed by center (*n* = 32)

	Centre 1	Centre 2	Centre 3	Centre 4	Centre 5
	*n* = 6	*n* = 7	*n* = 5	*n* = 7	*n* = 6
Female gender, No.	6	4	2	7	6
Age, years^a^ (range)	35 (27–59)	47 (44–75)	56 (47–61)	57 (32–61)	54.5 (31–63)
Current profession, No.					
(Pediatric) Oncologist/internist	1	5	4	1	3
Nurse	1	1	1	4	1
Other medical doctors (cardiologists, gynecologists)	‐	1	‐	1	1
Other HCPs (psychologists, physiotherapists)	3	‐	‐	1	1
Years of experience in pediatric oncology, years^a^ (range)	12 (5–34)	23 (19–51)	30 (20–33)	32 (10–40)	21 (8–39)
Number of CCSs seen in center per month, No.	20	170	18	75	200

^a^ Data are shown as medians.

### 3.2 TDF domains

**FIGURE 3:**
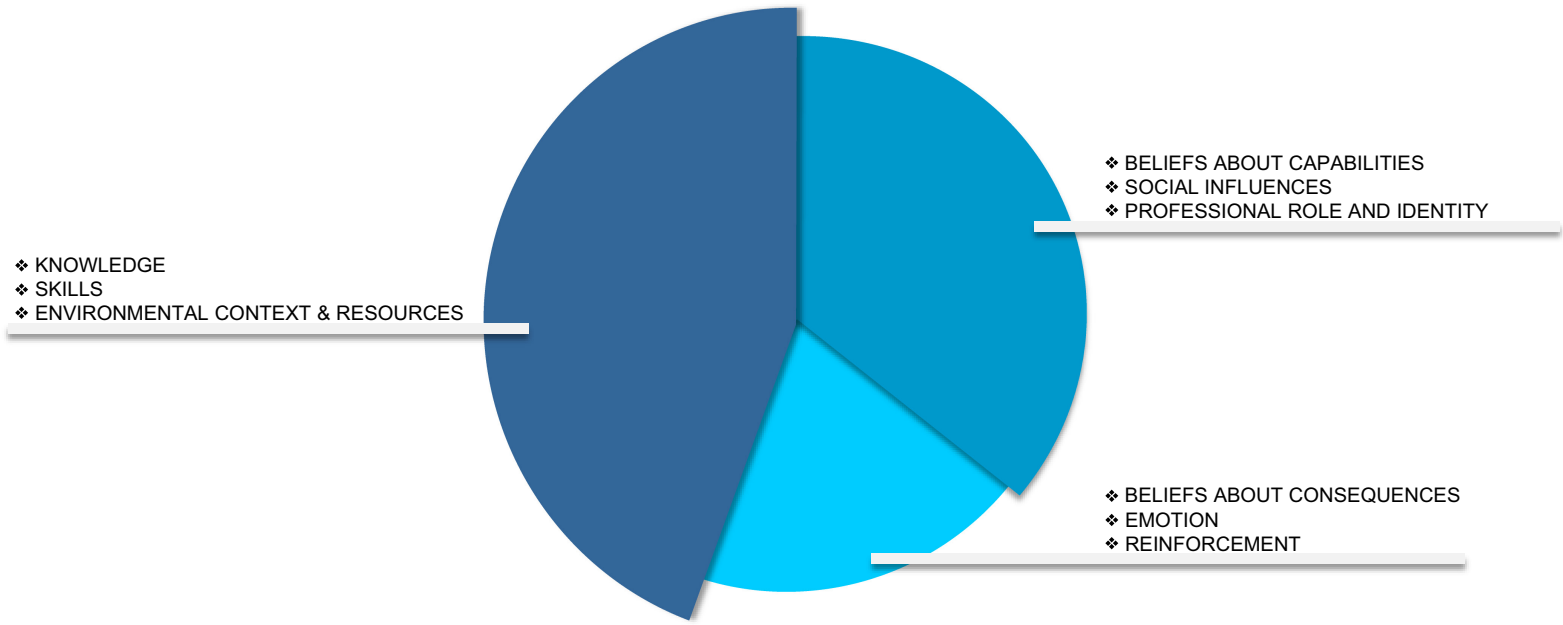
Relevant TDF domains regarding facilitators and barriers of HBS provision ordered by dominance in focus groups.

Nine out of the 14 TDF domains were identified in the data. Facilitators and barriers relating to the TDF domains “Knowledge”, “Skills” and “Environmental context and resources” were discussed by participants in all five focus groups and were therefore considered the most dominant TDF domains (Figure 3). The second most dominant domains were “Beliefs about capabilities”, “Social influences” and “Professional role and identity” (discussed in three to four focus groups). Lastly, “Beliefs about consequences”, “Emotion”, and “Reinforcement” were mentioned in two focus groups and therefore the least dominant. All relevant domains and the corresponding findings are summarized in Table 3. Illustrative quotes of the findings are presented in Table 4.

**TABLE 3:** Summary of key barriers and facilitators for providing HBS

Domain	Barrier	Facilitator
Knowledge	Lack of scientific evidence about benefits health behaviors for CCSs Lack of knowledge of late effects Lack of knowledge about healthy behaviors	Knowledge of importance of HBS in CCSs Knowledge of late effects Knowledge on how to pass on health information and to stimulate CCSs Knowledge about health behaviors
Skills	Lack of skills to motivate CCSs with HBS	Having the necessary skills to pass on information on health behaviors Having the necessary skills to identify right timing to raise issues on health behaviors Having the necessary skills to motivate CCSs
Social/professional role and identity	Sensitive nature of some health behaviors limits professional role of HCP Autonomous decisions of CCSs limits professional role of HCP Own responsibility of CCSs limits professional role of HCPs	Profession HCP important in HBS Specialty HCP important in HBS
Beliefs about capabilities	Lack of perceived competence in HBS Behavior patterns and addictions limiting capabilities of HCPs in HBS	‐
Beliefs about consequences	Belief CCSs themselves are a barrier for adopting and maintaining healthy behaviors Belief CCSs will not apply advice in real life	Belief education of CCSs by HCP will positively affect health behaviors in CCSs
Reinforcement	‐	Motivated CCSs act as positive reinforcement for HBS
Environmental context and resources*	Lack of good organizational structure for referring CCSs to other internal and external HCPs Barriers to implementation of HBS in current care Lack of available time	Good organizational structure for referring CCSs to other internal and external HCPs Good organizational structure relating to the outpatient clinic visit Available resources for HCPs to facilitate HBS Available informational resources to provide to CCSs Available resources for CCSs to stimulate exercise Available time for HBS
Social influences	‐	Health behavior discussions with other HCPs

* Dominant domain of the Theoretical Domains Framework based on high frequency.

**TABLE 4:** Illustrative quotes of barriers and facilitators to providing health behavior support

Domain	Quote
Knowledge	“I would appreciate having supportive evidence that it actually makes, […], if you are going to sell something, you need to not just go in and say, “Nobody should smoke,” but you can say it in a different way and say, “If you do this, you reduce your risk.”” (HCP #14, male, pediatric oncologist, center 3) “Or to have enough information on how we can pass it [health behavior information] on in a patient‐friendly way, […] so maybe something of a handle: how to transfer that in a breezy, understandable manner to all populations.” (HCP#2, female, nurse, center 1) “What can you say about the role of lifestyle in cancer survivors on the relative risk that the disease will come back? I do not have that knowledge and I find it very difficult to find.” (HCP #20, female, oncologist, center 4)
Skills	“To indicate in a directive way what is asked of them [survivors] gives a lot of results, because people themselves cannot oversee it well what it should be.” (HCP#22, female, nurse, center 4) “Time and sometimes also space. Sometimes they [survivors] are in the middle of a move, are people very busy or do they still work for 80 hours. Then you see there is no room at all and then sometimes after six months or so, the situation changes and then suddenly they start working on it or thinking about it.” (HCP#21, female, nurse, center 4).
Professional role and identity	“There are certain nurses, […], who are really focused on lifestyle and things like that. They can certainly give a different perspective in some other amount of time than the doctor has. I think the doctor's visit is usually quite medically focused. And we are not experts in lifestyle change.” (HCP #17, male, pediatric oncologist, center 3) “Well, it is with adults of course, who ‐ for example ‐ have been smoking for a long time, […], who am I to tell them to stop? But I always say something light‐hearted: “Gosh, I can say something about it when I'm wearing this white coat.”” (HCP #30, female, nurse specialist, center 5)
Beliefs about capabilities	“I would say that I'm comfortable in the knowledge of the effect of the different risk factors they have for developing heart disease, stroke, diabetes, all of this. But when it comes to specific advice, you have the motivated patients who want to do something about it, how to give that advice, I do not have any education in how to do that” (HCP #17, male, oncologist, center 3) “We think it's important, but we do not have the resources or the competencies most of the time” (HCP #14, male, pediatric oncologist, center 3) “The addiction aspect is very difficult to treat. With food addiction, alcohol, drugs, smoking. We always underestimate that.” (HCP #24, female, clinical psychologist, center 4)
Beliefs about consequences	“We really work towards insight, that they gain insight into “what has been my treatment, what is the risk?” and insight into “what is my lifestyle?” […]. That is the first approach, that's how we open it up and I also notice that the seed will grow, so that they eventually want to do something with it.” (HCP #21, nurse, center 4) “I think it's a shame when people have an enormous judgment about themselves, when something does not work out. That judgment stands in the way of success.” (HCP#28, internist, center 5) “It depends on the person. Some of them […] they are following and they are taking seriously what we recommend for them. But some of them, […], they are asking so many people, you know” (HCP #8, female, pediatric oncologist, center 2)
Reinforcement	“When someone says: “Yes, I actually have to lose weight, but it is very difficult.” Then you have an opening. Then I find it easier to talk about it than to say: “You should actually eat less or exercise more, or you should eat healthier.”” (HCP#30, nurse‐specialist, center 5)
Environmental context and resources	“I think the way in which we have structured the clinic, I also have the space to discuss that and to look at motivation and what someone has already done.” (HCP #20, female, oncologist, center 4) “We all draw our own experience from the past in relation to lifestyle advice, but it's not harmonized, and we need more discussions about that” (HCP #7, male, pediatric oncologist, center 2) “So, I think to have a lifestyle focus, you probably need to separate it and do it at a different time or by a different person in adjunction to the interview that they have with their physicians.” (HCP#15, male, oncologist, center 3) “Well, at first you just tell him to stop smoking. But he has probably heard that a hundred times. If someone is really like: “Now I really want to get started”, then it is a bit of searching like: “Where to can I refer someone like that?”.” (HCP #27, female, internist, center 5) “I think in tertiary hospitals with highly specialized care, it's [health behavior support] secondary to the treatment at hand or the follow‐up at hand. It's not very well implemented I think at this level.” (HCP #15, male, oncologist, center 2)
Social influences	“I notice that we really do learn from each other. […] You cannot know everything yourself, as long as you know where to go.” (HCP#29, oncology physiotherapist, center 5)

### 3.3 Knowledge

Knowing the importance of healthy behaviors in the CCSs population was indicated to facilitate HBS provision. However, a recurrent theme was that more evidence‐based knowledge on late effects and the influence of healthy behaviors on these late effects would benefit their practice. Being able to share detailed information on risk factors would be helpful to educate CCSs. A lack of proper knowledge on the relationship between unhealthy behaviors and late effects was indicated as a barrier for both (pediatric) oncologists/internists and nurses.

Concerns were expressed on providing knowledge without evidence as part of HBS in consultations. Some HCPs argued to wish having more knowledge on how to pass on information on health consequences of cancer and poor health behaviors to CCSs in a motivating manner. Besides, several HCPs indicated not feeling knowledgeable enough in advising CCSs on how they should adopt healthier behaviors. More education on what health behaviors encompass was therefore considered to be a enabling factor.

### 3.4 Skills

Participants expressed varied perspectives about the types of skills needed to provide effectively, information about the benefits of health behaviors. Factors likely to influence successful uptake of information about healthy behaviors were identified to lie with the CCSs and with the skills of the HCPs. The phase of life CCSs were in was considered to influence the uptake of information. Therefore, HCP skills in identifying the right timing for HBS was considered to be an enabling skill to improve the uptake of HBS. Skills in providing information with rationale, using a directive approach, were considered enablers for younger CCSs and those lacking any information about HBS. Another common view was a personalized approach helps to motivate CCSs. Mentioned examples of a personalized approach were making sure CCSs build trust in HCPs and will set achievable health behavior goals which fit in their daily routines.

### 3.5 Environmental context and resources

At organizational level, HCPs reported preferring a structure in which it is easy to refer CCSs to programs or other HCPs with more relevant competencies for HBS (e.g. dieticians and physiotherapists). Some HCPs argued to experience difficulties in their current organizational structure to refer CCSs as it was unclear whom to refer to. Another reported organizational barrier was implementation of HBS in tertiary care, as it is often seen as a primary care matter. A good structure of follow‐up visits for CCSs at survivorship care clinics was believed to be a facilitator. By arranging the structure in such a way that during a clinic visit, health behaviors can be discussed with HCPs upon entry, during and after the consultation, HCPs will be able to have more in‐depth health behavior discussions with CCSs, rather than only signaling unhealthy behaviors. Concerning resources, HCPs believed harmonized guidelines could improve the content of HBS. Both a survivor care plan, as well as brochures with health behavior tips, were mentioned as examples of facilitating informational resources for CCSs. Some HCPs reasoned CCSs should be offered rehabilitation programs to stimulate exercise. A recurrent theme was available time for HBS. Whilst a minority mentioned perceiving the time available to discuss health behaviors as sufficient, most HCPs considered their dedicated time for HBS as insufficient as other health problems of CCSs had often more priority to discuss. Other HCPs attributed the lack of time to be related to the available financial resources of the organization to commit to HBS and to arrange enough personnel to provide HBS in a proper manner.

### 3.6 Professional role and identity

Specialties and professions of HCPs were commonly viewed to be significant factors for successful HBS provision. HCPs such as lifestyle coaches or general practitioners were, considering their knowledge of health behaviors, perceived as good examples of professions with higher odds of successful HBS. Besides, nurses were believed to be more influential on CCSs than doctors. Concerns about their own professional role as HCPs in HBS were related to the sensitive nature of specific health behaviors including alcohol and drug use, the freedom for CCSs to make own decisions, and own responsibility for CCSs in health behaviors.

### 3.7 Beliefs about capabilities

Regarding their perceived HBS capabilities, feeling that their competence was lacking was a barrier for HCPs. This competence was negatively influenced by the difficulty of changing behavior patterns and addictions of CCSs. Another barrier for HBS was related to the negative perception of HCPs about the success of such support.

### 3.8 Beliefs about consequences

Some HCPs believed that CCSs will not apply their advice in real life as only advice provision may not be enough for CCSs. While a few HCPs believed education by HCPs to be an important contributor to successful health behavior adoption of CCSs, others believed the personal situation and problems of CCSs might obstruct effective health behavior change.

### 3.9 Reinforcement

Concerning their incentives to provide HBS, some HCPs argued to be triggered by CCSs who are already motivated to change their health behaviors. Though sometimes obstructed by difficulties to provide HBS, HCPs felt obliged to give their best efforts to search for the right HCP to refer CCSs to. In addition, motivated CCSs made it easier for HCPs to start in‐depth health behavior conversations with CCSs.

### 3.10 Social influences

Regarding the interactions of HCPs have with others that may influence HBS provision, HCPs mentioned discussions with co‐workers on HBS in CCSs as facilitating to HBS provision. HCPs indicated to learn a lot from other co‐workers by having those conversations during, for instance, patient consultations in which a particular case of a survivor is discussed.

### 3.11 HBS provision by survivorship care center

**FIGURE 4:**
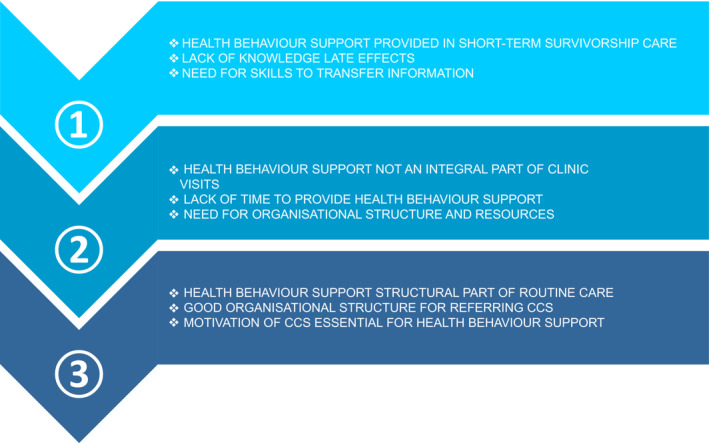
Summary of facilitators and barriers to providing HBS as perceived by HCPs affiliated with centers in different phases of HBS implementation in current care.

Facilitators and barriers mentioned by HCPs were also related to the implementation phase of HBS for long‐term CCSs in a survivorship care center (Figure 4). Three distinct phases of HBS implementation in current care were identified from the focus groups: (1) HBS mainly provided to short‐term CCSs; (2) HBS not structurally provided to long‐term CCSs; (3) HBS provided as part of usual care to long‐term CCSs. HCPs affiliated with center 1 indicated that, at time of the study, HBS was mostly provided to short‐term CCSs; i.e., CCSs less than 5 years off therapy. They were working towards more survivorship care provision to long‐term CCSs in their center. As their center mainly focused on early survivorship care with attention to cancer recurrence, knowledge on long‐term health effects of childhood cancer was limited. However, they did perceive HBS as essential to the support recovery of childhood cancer patients and expressed a need for skills to pass on information. Most HCPs of phase two, including centers without structural HBS provision to CCS (centers 2 and 3), recognized the importance of HBS, but as it was not an integral part of clinic visits of CCSs, the approach of HBS differed on an individual basis. A structured organization was lacking at these centers and time for HBS was sometimes a limiting factor. At phase 3 (including centers 4 and 5), where HBS had been fully implemented, health behaviors were structurally discussed during clinic visits of CCSs and the organizational structure allowed HCPs to refer CCSs to internal or external HCPs with expertise in health behaviors. Here, HCPs were mostly concerned with the motivation level of CCSs, which was often reported to be an essential factor for successful HBS in CCSs. Moreover, most HCPs at phase 3 believed that their role was to support CCSs in taking personal control in health behavior change.

## 4 DISCUSSION

This is the first study to identify facilitators and barriers of health behavior support (HBS) provision to childhood cancer survivors (CCSs) as perceived by healthcare professionals (HCPs) affiliated with different centers across Europe. Overall, this study showed that facilitators and barriers were most frequently related to the TDF domains “Knowledge”, “Skills”, and “Environmental context and resources”. Key findings suggest that having sufficient knowledge about health behaviors, health behavior interactions with late effects, and health behavior communication are important facilitators of HBS provision by HCPs. In addition, having health behavior communication skills are beneficial. At an organizational level, this study found that a good structure for referral of CCSs to other HCPs and having an efficient clinic visit could stimulate HBS provision. Lastly, lack of time was perceived as a major barrier in providing HBS.

In our study, knowledge about survivors' risks of developing late effects, and what health behavior(s) can contribute to these, was found to be suboptimal in HCPs and could therefore be enhanced. Current scientific evidence on the necessity to promote healthy behaviors in CCSs was perceived as insufficient. Previous studies also confirmed a lack of knowledge about health behaviors in HCPs working in the adult oncology setting.^18^, ^28^ Though HCPs in the current study expressed a need for harmonized guidelines as a resource to be used in clinical practice, HCPs may not be aware of the current guidelines supporting healthy behaviors in CCSs.^10^, ^29^ For instance, the International Guideline Harmonization Group for late effects of childhood cancer has published literature‐based recommendations for physical activity in survivors treated with anthracyclines or chest radiation.^29^ Scientific meetings could serve as good ways to disseminate this existing knowledge about health behaviors in CCSs to HCPs working in the survivorship care setting. For further knowledge and skills improvement, it is important to explore current curricula of HCPs working with CCSs and if proven to be insufficient, to offer HCPs more training and educational opportunities on health behaviors in CCSs. To limit time, travel‐, and carbon emissions burden, e‐courses or in‐house training led by other HCPs integrated in the organization could be considered.

Another important finding was that HCPs perceived acquiring soft skills regarding health behavior education towards CCSs as favorable in HBS. In a study by Hardcastle et al., oncologists similarly indicated that they perceived a lack of training or guidance in exercise prescription for cancer survivors to be an obstacle in promoting physical activity.^30^ Training, for instance in person‐centered care, to enhance those skills could provide HCPs the techniques needed for adequate HBS.^31^ By taking the survivor's personal preferences and values into account, person‐centered care could serve as a means for better health behavior communication with CCSs. The approach, based on partnership between HCPs and patients, shared‐decision making, and empowerment of patients, could make CCSs more receptive to health behavior information.

The current study found that organizational structure plays a major role in the provision of HBS. Having a structured survivorship care clinic with a network of internal and external HCPs to refer CCSs to is essential for centers. This finding is in accordance with a study by Keogh et al. in which oncology nurses perceived the lack of adequate support structures as barriers to physical activity promotion.^32^ At an organizational level, centers should therefore work on establishing networks with both secondary and primary care.

Consistent with this study, Koutoukids et al. also reported a lack of time to be a barrier to lifestyle advice provision by HCPs working with cancer survivors.^28^ Having a structured outpatient clinic visit may solve this barrier. This could be accomplished by allowing health education to be part of routine follow‐up visits of CCSs. Implementing a new organizational structure requires effort and can potentially lead to further needs in terms of human and materials resources as well as to a reallocation of resources. Nevertheless, considering the differences observed in the respective centers that have participated in the study we can say that such changes facilitate HBS for CCSs.

Although the present study gives a unique insight into the facilitators and barriers of HBS provision to CCSs as perceived by HCPs, some minor limitations should be noted. Firstly, this study also included HCPs with a supportive role in survivorship care such as physiotherapists, cardiologists, and psychologists with not all seeing CCSs on a daily basis. However, as this only applied to 25% of the participating HCPs, this limitation does not influence the results. Besides, including a variety of HCPs gives perspectives of all kinds of HCPs working in the survivorship care setting. Secondly, due to limited resource availability, we were only able to conduct focus groups with survivorship care centers participating in the PanCareFollowUp project. Therefore, though we are of the opinion that our study had sufficient information power and participants in total (*n* = 32), the sample size of 5 focus groups was decided beforehand and including more centers to reach full data saturation was not possible. Thirdly, due to a lack of male healthcare professionals involved in survivorship care at three out of the five participating centers, overall, the male gender was underrepresented in the study. This could potentially have influenced the results as male healthcare professionals may experience other barriers or facilitators when providing health behavior support. Fourthly, due to the international character of this study, two out of the five focus groups could not be conducted in the native language of the participants. Therefore, as sufficient English language skills were part of the inclusion criteria, this may have excluded some HCPs. However, given the composition of the focus groups, we still believe they were representative for this study. Lastly, HBS is not composed of a single unit of behavior, but is rather a series of actions delivered by HCPs; i.e. screening for unhealthy behaviors and informing and advising CCSs about adopting healthier behaviors. As the TDF is meant for singular behaviors, the facilitators and barriers could have potentially been different per action.

## 5 CONCLUSIONS

Taken together, these results suggest that big opportunities exist for education and training of HCPs to increase their competencies for HBS and for centers to structure their clinic visits and expand referral options. Though it might be challenging, survivorship care clinics should also work to establish well‐integrated care with HBS being part of routine care. Further understanding of facilitators and barriers reported in this study will inform HCPs about the implementation of HBS in survivorship care of CCSs.

## AUTHOR CONTRIBUTIONS

**Eline Bouwman:** Conceptualization (equal); data curation (lead); formal analysis (lead); investigation (lead); methodology (equal); project administration (equal); visualization (equal); writing – original draft (lead). **Saskia M.F. Pluijm:** Conceptualization (lead); funding acquisition (equal); project administration (supporting); supervision (equal); writing – review and editing (lead). **Iridi Stollman:** Formal analysis (lead); investigation (equal); validation (equal); visualization (lead); writing – review and editing (supporting). **Vera Araujo‐Soares:** Conceptualization (lead); funding acquisition (supporting); methodology (equal); writing – review and editing (lead). **Nicole M.A. Blijlevens:** Supervision (supporting); writing – review and editing (supporting). **Cecilia Follin:** Project administration (equal); resources (lead); writing – review and editing (supporting). **Jeanette Falck Winther:** Funding acquisition (equal); resources (equal); writing – review and editing (supporting). **Lars Hjorth:** Funding acquisition (equal); resources (equal); writing – review and editing (equal). **Tomas Kepak:** Funding acquisition (equal); resources (equal); writing – review and editing (supporting). **Katerina Kepakova:** Project administration (equal); resources (lead); writing – review and editing (supporting). **Leontien C.M. Kremer:** Funding acquisition (lead); writing – review and editing (equal). **Monica Muraca:** Funding acquisition (equal); writing – review and editing (supporting). **Helena J.H van der Pal:** Funding acquisition (equal); project administration (equal); resources (lead); writing – review and editing (equal). **Carina Schneider:** Writing – review and editing (supporting). **Anne Uyttebroeck:** Funding acquisition (equal); project administration (equal); resources (lead); writing – review and editing (supporting). **Gertrui Vercruysse:** Writing – review and editing (supporting). **Roderick Skinner:** Conceptualization (equal); funding acquisition (equal); writing – review and editing (lead). **Morven C. Brown:** Conceptualization (equal); funding acquisition (equal); methodology (equal); writing – review and editing (lead). **Rosella P.M.G. Hermens:** Funding acquisition (equal); methodology (lead); project administration (supporting); supervision (lead); validation (equal); writing – review and editing (lead). **J.J. Loonen:** Conceptualization (lead); funding acquisition (equal); project administration (equal); resources (lead); supervision (lead); validation (equal); writing – review and editing (lead).

## FUNDING INFORMATION

This work was supported by the European Union's Horizon 2020 Framework Program [grant number *824982*]. The funder had no role in study design, data collection, data analysis, data interpretation, or in writing the report. The material presented and views expressed here are the responsibility of the author(s) only. The EU Commission takes no responsibility for any use made of the information set out.

## CONFLICTS OF INTEREST

The authors declare that they have no known competing financial interests or personal relationships that could have appeared to influence the work reported in this paper.

## ETHICAL APPROVAL STATEMENT

The procedures of this study were approved by METC Oost‐Nederland (case number 2019–5630) and conforms to the principles of the Declaration of Helsinki (Fortaleza, Brazil, Version 2013) and the Dutch Medical Research Involving Human Subjects Act.

## PATIENT CONSENT STATEMENT

Written informed consent was obtained from all participants.

## Supporting information


Table S1.

## Data Availability

The PanCareFollowUp project aims to comply with all the four FAIR principles and to share individual de‐identified data upon request. At moment of writing, both the PanCareFollowUp project and the Radboud University Medical Centre are working on establishing the conditions and means to share the data. Requests for de‐identified data should be made to the corresponding author (EB).
