# Supplementary material for: Healthcare professionals' perceived barriers and facilitators of health behavior support provision: A qualitative study
Source: Cancer Med. 2022 Nov 17;12(6):7414–26. doi: 10.1002/cam4.5445 (PMC10067039; doi:10.1002/cam4.5445)
Supplement: Supplementary file 1 — Table S1. [file CAM4-12-7414-s001.docx]

**Supporting Information**

**Table S1**. Topic Guide Focus Groups Healthcare Professionals

| **Topic/domains** |  | **Questions** | **Probes** |
| --- | --- | --- | --- |
| *Delivery of lifestyle advice in general* |  | 1. *To what extent is lifestyle discussed when they visit the clinic?* 2. *Are you providing lifestyle advice to survivors coming to the clinic as part of routine care? In case yes, can you tell me something about how you are dealing with providing lifestyle advice? Prompt: is it structured?* 3. *Are you providing lifestyle advice to survivors coming to the clinic as part of routine care? In case yes, can you tell me something about how you are dealing with providing lifestyle advice? Prompt: is it structured?* |  |
| *General believes and behaviors providing lifestyle advice* |  | 1. *How do you think/feel about delivering lifestyle advice to survivors? Prompt: thoughts and views* 2. *What kind of things hinder you/your co-workers in providing lifestyle advice to (childhood) cancer survivors (probe: barriers)?* 3. *What kind of things help you in providing lifestyle advice to (childhood) cancer survivors (probe: facilitators)?* |  |
| *Knowledge* |  | 1. *What do you know about the importance of a healthy lifestyle for this specific population (cancer survivors)?* 2. *Are there any specific guidelines used in the late effect clinic? In case yes, are these guidelines appropriate for the childhood cancer survivor population?* | - Physical activity - Diet - Smoking behaviors - Alcohol/ drug use |
| *Professional role and identity* |  | 1. *How do you think about your responsibility as a health care professional in your function to provide the survivor lifestyle advice?* 2. *What do you think about the responsibility of other HCPs to provide lifestyle advice to advisors? (prompt - do you think all HCPs are equally responsible for giving lifestyle advice? Explain)* | - Professional identity - Responsibilities |
| *Skills, beliefs about capabilities and emotions* |  | 1. *Can you tell me something about your competence to give lifestyle advice to survivors?* 2. *How confident are you confident that you can deliver lifestyle advice to your survivors that really comes through? Explain more why you are or aren’t confident.* 3. *How comfortable are you in delivering lifestyle advice to survivors?* | - Communication skills - Competence - Self-confidence - Perceived competence - Self-efficacy - Empowerment |
| *Environmental context and resources* |  | 1. *Can you tell me something about the time you have during face-to-face contact with survivors to give lifestyle advice? Is this enough in your opinion?* | - Resources (time) |
| *Social influences* |  | 1. *How do you think other co-workers or other HCPs feel about providing lifestyle advice to survivors?* 2. *Do you ever discuss issues on providing lifestyle advice to survivors with your co-workers or other HCPs?* 3. *How are you influenced by media or the scientific world about lifestyle advice?* | - Social pressure - Social comparisons - Social support - External pressure (media/scientific world) |
| *Memory, attention, and decisions process* |  | 1. *In case you are providing lifestyle advice as part of standard care, can you tell me something about your steps in decision-making to provide lifestyle advice to survivors? Which survivor characteristics are important in this process?* | - Decision-making |
| *Optimism* |  | 1. *How optimistic are you that your lifestyle advice to survivors will trigger/help the survivor in changing his/her lifestyle?* | - Optimism - Pessimism |
| *Behavioral regulation* |  | 1. *What do you think is necessary for you and/or your co-workers to make providing lifestyle advice part of standard follow-up care for survivors? (Or to keep it feasible for you to maintain providing lifestyle advice)* |  |
|  |  |  |  |
| **Closing**   \| Is there anything else you would like to add about providing lifestyle advice to survivors? \| \| --- \| | | | |
